# Evaluation and management of anterior urethral stricture disease

**DOI:** 10.12688/f1000research.7121.1

**Published:** 2016-02-09

**Authors:** Altaf Mangera, Nadir Osman, Christopher Chapple

**Affiliations:** 1 Department of Urology Research, Royal Hallamshire Hospital, Sheffield, UK

**Keywords:** Urethral stricture disease, urethrotomy, dilatation, urethrography

## Abstract

Urethral stricture disease affects many men worldwide. Traditionally, the investigation of choice has been urethrography and the management of choice has been urethrotomy/dilatation. In this review, we discuss the evidence behind the use of ultrasonography in stricture assessment. We also discuss the factors a surgeon should consider when deciding the management options with each individual patient. Not all strictures are identical and surgeons should appreciate the poor long-term results of urethrotomy/dilatation for strictures longer than 2 cm, strictures in the penile urethra, recurrent strictures, and strictures secondary to lichen sclerosus. These patients may benefit from primary urethroplasty if they have many adverse features or secondary urethroplasty after the first recurrence.

## Introduction

This update will concentrate on the advances in the investigation and management of urethral stricture disease in men. Traditionally, urologists have offered such men urethral dilatation/urethrotomy, which carries the risk of needing repeat interventions and a long-term need to self-dilate the urethra on a regular basis. We aim to discuss the evidence relating to patient investigation and selection for more definitive surgery such as urethroplasty.

A urethral stricture is a narrowing of the urethra. A “true” stricture is the result of ischaemic spongiofibrosis manifesting as scar tissue in the corpus spongiosum ^1^. Contraction of this scar tissue leads to a reduction in the urethral calibre, which leads to voiding difficulty. On the other hand, urethral distraction injuries occur as a result of blunt trauma distracting the two ends of the urethra apart and are not “true” strictures. Ischaemic spongiofibrosis may be due to infection such as gonococcal urethritis, inflammation such as lichen sclerosus, or instrumentation; however, the majority of strictures are idiopathic. It is reported that, in the US, on the basis of 10 public and private databases between 1992 and 2000, there were 5 million office visits per year and more than 5,000 inpatient admissions per year due to urethral strictures ^2^.

There are many management options available for treating urethral stricture disease, commencing with less invasive urethral dilatation, urethral stenting and urethrotomy, and progressing to anastomotic and augmentation urethroplasty. The optimum management approach is often debated by urologists; some prefer a less invasive approach and perform urethrotomy/dilatation as first and even second line for all patients, whereas those who perform urethroplasty regularly recognise that urethroplasty may become more difficult after urethrotomy and advocate primary urethroplasty, as it is thought that a urethrotomy lengthens the stricture and leads to deepening of the spongiofibrosis, resulting in poorer blood supply to the urethra. Also, in a multivariate analysis of urethroplasty outcomes, prior urethrotomy was found to be a risk factor for failure ^3^. Others take a “middle of the road” approach and select patients in whom urethrotomy is likely to have a limited role and counsel them regarding primary urethroplasty. In this article, we aim to review the literature regarding the investigation and selection of patients for urethroplasty.

## Discussion

### Stricture evaluation

Much work has gone into providing a means of identifying the extent of a urethral stricture pre-operatively. Traditionally, a retrograde urethrogram is used to identify stricture density and length. Ideally, an antegrade and retrograde urethrogram could be performed to fully characterise the stricture. A recent study has suggested that the operating urologist may be better off performing and interpreting the urethrogram, as this led to the most accurate finding of strictures and description of stricture length ^4^. In this study, all urethrograms were performed by a urologist and therefore it is not known whether the radiologist would have obtained the same results had they performed and reported the studies. Certainly, in our practice, a sub-speciality uro-radiologist performs and reports the study and it is thereafter viewed by the operating surgeon.

Another method of stricture assessment involves the use of ultrasound. This can accurately assess the extent of ischaemic spongiofibrosis in the corpus spongiosum and this is often longer than the “white” stricture which is seen at endoscopy, which in turn is longer than the “narrowing” seen on the urethrogram ^5^. A recent report from McAninch *et al*. has shown that this can pick up underlying spongiofibrosis, which changed the stricture length in 45% of patients over a urethrogram ^6^. In this series of 232 men, the urethroplasty approach was changed in 19% of patients. Strictures which appear short on urethrography but have extensive underlying spongiofibrosis are important to identify as these are more likely to require substitution urethroplasty instead of anastomotic urethroplasty. In the series from McAninch *et al*., the mean stricture length was increased by the use of ultrasonography from 2 to 3.4 cm. Another study, of 40 patients, has suggested that ultrasound is accurate in assessing anterior urethral strictures and also provides more information than the urethrogram alone ^7^. Ultrasound has also been found to be equivalent to magnetic resonance urethrography and the latter is probably unnecessary for assessing the anterior urethra ^8^.

Another important use for ultrasound, which has not been investigated, is in deciding which patients are likely to benefit from a urethral dilatation/urethrotomy or will likely require a urethroplasty. It is thought that by incising a stricture, the underlying fibrosis is lengthened and thus subsequent stricturing, if it recurs, is likely to lead to a longer stricture. In an interesting retrospective study, it was shown that bulbar strictures where urethrotomy/dilatation had been undertaken two or more times were longer and also recurred quicker than in those who underwent one or no transurethral surgery ^9^. The difficulty here is that we have “the cause or effect scenario” as it cannot be proven, in this study, whether the strictures undergoing more transurethral surgery were longer and therefore more apparent symptomatically to begin with.

The capability of a urethra to heal without re-stricturing is reliant upon an adequate underlying blood supply and therefore knowledge of underlying spongiofibrosis may well be beneficial in identifying those patients who may benefit from primary urethroplasty. A recent Société Internationale d’Urologie/International Consultation on Urological Diseases (SIU/ICUD) consultation for evaluation and follow-up of urethral stricture disease concluded that urethrography and urethroscopy remain the investigations of choice for the anterior urethra ^10^. The evidence for the use of ultrasound is currently limited but does show some promise in evaluating underlying spongiofibrosis and may help counsel patients better before undertaking surgery as to the type of surgery they may require.

### Stricture management

Traditionally, the most commonly performed procedure for urethral stricture has been dilation/urethrotomy. A survey of 1,262 American urologists found that most urologists treat between 6 and 20 strictures per year and over 90% performed dilatation/urethrotomy ^11^. It is noteworthy that 74% of urologists believed that urethroplasty should be performed only after repeated failure of endoscopic methods.

A randomised controlled trial reported by Steenkamp *et al.* reviewed 210 men, of whom 106 underwent dilatation and 104 urethrotomy ^12^. At 1 year, there was a success rate of 60% if the stricture was less than 2 cm, 50% if it was between 2 and 4 cm, and 20% if it was more than 4 cm in length. In a subsequent publication, Heyns *et al*., working on the same dataset, looked at repeat dilatation/urethrotomy and noted that after a single treatment 70% would be stricture-free at 3 months, 35–40% would remain stricture-free at 48 months, and a secondary procedure was of limited benefit at 24 months, but not at 48 months ^13^. A third treatment was of no benefit at all. Other authors have reported even worse success rates, with primary urethrotomy approaching even less than 40% ^14, 15^. However, these are retrospective case reports and caution should be applied when interpreting their findings.

Greenwell *et al*. concluded that, in terms of cost-effectiveness, the use of urethroplasty after failure of one urethrotomy/dilatation was likely to be the most cost-effective approach ^16^. Contrastingly, Rourke and Jordan have suggested that treatment of short bulbar strictures by urethroplasty is more cost-effective than urethrotomy ^17^. A calculated approach is that initial urethrotomy followed by urethroplasty is the most cost-effective approach if there is recurrence of the stricture, unless the success rate of urethrotomy was likely to be inferior to 35% ^18^. Certainly, experts who perform urethroplasty regularly do feel it is made more difficult by repeated interventions such as urethrotomy. A retrospective review by Roehrborn and McConnell reported doubling of the failure rate in patients with previous surgical manipulation ^19^. Similarly, Breyer *et al*. reported a hazard ratio of 1.7 on multivariate analysis of 443 patients if they had previously undergone urethrotomy ^3^.

The reasons some urologists offer repeat urethrotomy are manifold. A recent study of case logs from the US showed great disparity in the number of urethroplasties performed in different regions, and men were more likely to be referred for specialist intervention by newly certified urologists than established urologists ^20^. All urologists are familiar with urethrotomy/dilatation and therefore are more likely to offer this than refer to another institute where urethroplasty is performed regularly. In addition, patient comorbidities may exclude a patient from having urethroplasty ^21^. Similar findings were reported by a European study, in which 79% of Dutch urologists reported that they felt a urethroplasty should be offered only after failed urethrotomy ^15^. The authors found that 20% of urologists would continue to perform urethrotomy for a 1 cm stricture even after two recurrences. Therefore, it transpires for some patients that urethroplasty is not considered at all or considered only when the stricture is lengthy and subsequent urethroplasty more difficult.

Table 1 lists the factors which should be considered as risk factors for recurrence of urethral strictures ^22^. In our experience, bulbar strictures are likely to recur less often after urethrotomy than penile urethral strictures because of better blood supply in the bulbar urethra, although there are limited data on this in the literature. Strictures which recur after urethrotomy/dilatation are almost certainly likely to need further intervention after a repeat urethrotomy/dilatation. We feel that by assessing these in each individual patient, three categories of patients can be created. Those with multiple risk factors should proceed to urethroplasty if suitable, and those with two risk factors may undergo primary urethrotomy/dilatation but should be counselled regarding urethroplasty. Finally, those with only one risk factor could undergo urethrotomy/dilatation first. This approach, though useful in clinical practice, does require some fine tuning, and only with emerging evidence will it be possible to give a weighting to each risk factor.

**Table 1.** Factors important for urethral stricture recurrence.

Factors	Good prognosis	Poor prognosis
Length	<2cm	>2cm
Location	Bulbar urethra	Penile urethra
Aetiology	Idiopathic	Inflammatory, Iatrogenic Lichen sclerosus
Recurrent	No	Yes

### Follow-up

Many surgeons will rely upon the visual appearance of the urethra at cystourethroscopy; an ischaemic urethra looks white or grey, and healthy well-vascularised tissue appears pink. The narrowed portion of the urethra may appear much shorter than the white area with underlying spongiofibrosis. Cystourethroscopy provides earlier evidence of stricturing or recurrence usually prior to a reduction in flow rate ^23^. The flow rate is not significantly affected until the urethral calibre is less than 11 Fr ^24^. In our experience, we advocate symptom assessment and cystourethroscopy for follow-up. The frequency and length of follow-up should follow a risk-stratified approach as discussed above for patient assessment ( Table 1). The evidence for this approach, however, is currently lacking in the literature.

Patients with strictures may also present with a multitude of symptoms which may or may not impact upon their quality of life ^25^. In this regard, a Patient-Reported Outcome Measure (PROM) has been validated for this cohort ^26^. This also takes account of sexual function and should be used in the assessment of patients pre- and post-operatively. A more recent report has shown this PROM to be able to detect post-operative changes after 2 years of follow-up ^27^.

### Future work

Questions remaining for the future include the use of ultrasound in deciding which options are beneficial for patients prior to the first urethrotomy or for urethroplasty. Further investigation is required into factors that lead to lower success with urethrotomy, which are also those leading to worse outcomes with urethroplasty. The results of the Open urethroplasty versus Endoscopic urethrotomy (OPEN) randomised controlled trial are eagerly awaited. This specifically looks at the use of urethroplasty or urethrotomy for recurrent strictures. A Cochrane review has shown the dearth of randomised controlled data in this field and these are what we really require ^28^. Depending on the findings of the OPEN trial, the field should be able to progress in the correct direction and lead to better patient selection and, ultimately, improved patient outcomes.

## Conclusions

From the discussion above, it is clear that proper assessment of urethral strictures is required to dictate appropriate management. All too often, a stricture is seen via flexible cystoscopy and the patient placed on the waiting list for a urethrotomy which is left to a junior resident to “cut through”. Surgeons should appreciate that by cutting through a stricture they may be lengthening the stricture as it relies on an adequate residual blood supply to heal without scarring. It should be recognised that the success of their intervention is limited by certain stricture characteristics and not all strictures are the same. Variations in strictures should be noted and respected. Stricture length, location and aetiology have been shown to affect recurrence rate, and urologists should be able to classify strictures as high-, intermediate- and low-risk ( Table 1). With these factors in mind, a urologist should be more equipped in counselling patients regarding their options for management and follow-up.

## Abbreviations

OPEN, Open urethroplasty versus Endoscopic urethrotomy; PROM, Patient-Reported Outcome Measure.
